# The role of peri-aortic fat in aortic atherosclerosis

**DOI:** 10.1186/1532-429X-11-S1-O58

**Published:** 2009-01-28

**Authors:** Ilias Kylintireas, Ikhlef Bechar, Cheerag Shirodaria, Alistair Lindsay, Justin Lee, Matthew Robson, Stefan Neubauer, Robin Choudhury

**Affiliations:** University Of Oxford, Oxford, UK

**Keywords:** Visceral Adipose Tissue, Plaque Index, Turbo Spin Echo, Aortic Distensibility, Aortic Atherosclerosis

## Introduction

Recent data suggest that perivascular fat is metabolically active and may play a role in the initiation and progression of atherosclerosis mediated by the paracrine action of the adipokines it produces. MRI offers the unique capability for clear depiction and accurate quantification of adipose tissue alongside an effective non invasive and radiation free assessment of both early and advanced atherosclerotic effects on the vasculature.

## Purpose

We investigated the relationship between perivascular adipose tissue and atherosclerosis-related structural and functional changes of the vasculature in an elderly population at high cardiovascular risk.

## Methods

Fifty elderly subjects [mean age = 65 (± 8), 16/50 = 32% women] with at least one major cardiovascular risk factor (smoking, diabetes, hypertension, hyperlipidemia) underwent MRI (1.5 T Siemens Sonata) for abdominal and peri-aortic adipose tissue quantification and aortic atherosclerosis assesment. A Water Suppression (WS) T1 weighted (T1W) Turbo Spin Echo (TSE) multi-slice sequence was used for visceral adipose tissue (VAT) and abdominal subcutaneous adipose tissue (SCAT) measurement.

**Figure 1:**
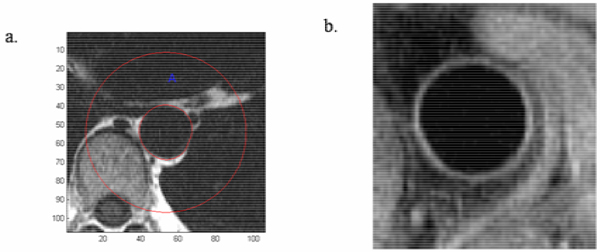
Figure 1

A modified version of the same sequence was used to produce WS cross-sectional images covering the descending thoracic aorta (figure 1a). On these images peri-vascular adipose tissue (PVAT) area was measured within a radius equal to three times the radius of the vessel cross-section. PVAT index (PVATI) was defined as the PVAT area divided by the cross-sectional area of the vessel (in order to normalise to the vessel size) and averaged for all the images along the aorta to produce a PVATI value per patient. Inter-scan and intra-observer variability of this method was very satisfactory (coefficient of variance was 0.06 and 0.03 respectively).

Proton density weighted fat saturation black blood turbo spin echo (TSE) cross-sectional images covering the descending thoracic aorta (figure 1a) were used for atheroma burden measurements (expressed as plaque index (PI) = cross-sectional vessel wall area/total cross-sectional vascular area).

Aortic distensibility was assessed from breath-hold ECG-gated, steady state free precession (SSFP) images through the thoracic descending aorta. Distensibility was calculated as the relative change in area divided by peripheral pulse pressure.

## Results

**Figure 2:**
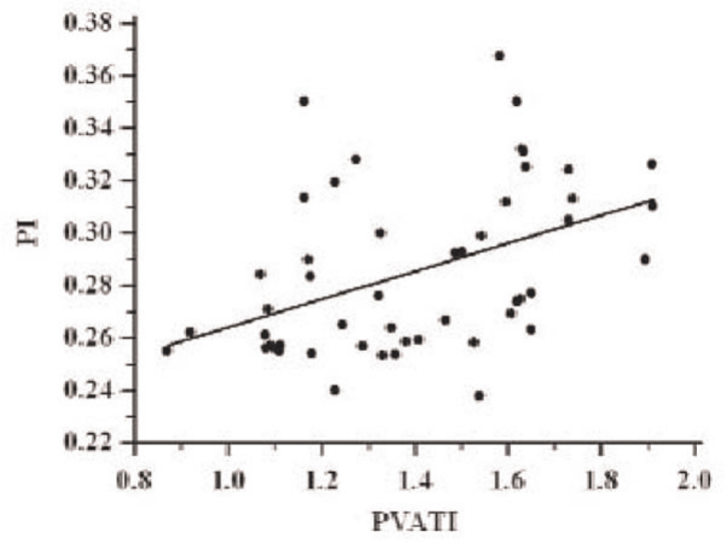
Figure 2

**Figure 3:**
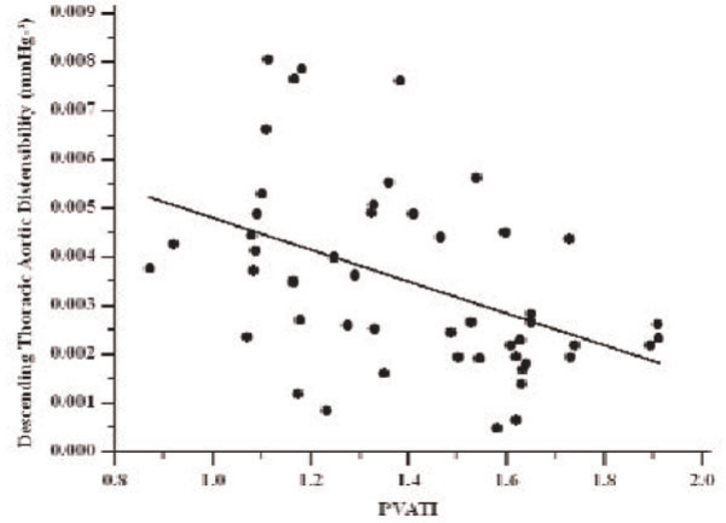
Figure 3

Aortic PVATI correlated with aortic atheroma burden (r = 0.44, P < 0.005) (figure 2) and inversely correlated with descending thoracic aortic distensibility (r = -0.45, P < 0.005) (figure 3). VAT and SCAT measurements did not correlate with thoracic aortic PI or descending thoracic aortic distensibility. Applying corresponding multiple regression analysis models (including classical risk factors, demographics and anthropometric measurements) PVATI emerged as an independent predictor of both aortic atheroma burden [b = 0.06 (± 0.02), P < 0.05, R^2^ = 0.35] and descending thoracic aortic distensibility [b = -0.00014 (± 0.00005), P < 0.01, R^2^ = 0.42].

## Conclusion

These results suggest a pathophysiological link between peri-vascular adiposity and the atherosclerotic process in the underlying vessel. MRI is an effective tool in depicting and quantifying PVAT. Further, interventional and analytical studies are required to elucidate this relationship.

